# Lectin-Glycan Interactions in Corneal Infection and Inflammation

**DOI:** 10.3389/fimmu.2018.02338

**Published:** 2018-10-08

**Authors:** Dina B. AbuSamra, Pablo Argüeso

**Affiliations:** Department of Ophthalmology, Schepens Eye Research Institute of Massachusetts Eye and Ear, Harvard Medical School, Boston, MA, United States

**Keywords:** cornea, galectin, glycosylation, infection, inflammation

## Abstract

The cornea is an extraordinary component of vision that functions as the principal barrier to pathogens in the eye while allowing light transmission into the retina. Understanding the cellular and molecular mechanisms that maintain homeostasis in this tissue is the subject of intense scientific study given the high prevalence of corneal disease. Over the past decade, the interactions between lectins and glycans on plasma membranes have emerged as important regulatory factors in corneal biology. In particular, members of the galectin family have been shown to bind multiple β-galactoside-containing receptors to regulate immunopathological processes associated with viral and bacterial infection, transplantation, wound healing, dry eye, angiogenesis, and lymphangiogenesis. In this review, we describe the current understanding of how these surface interactions intersect with different pathways to activate unique cellular responses in cornea as well as their potential therapeutic implications.

## Introduction

Lectins are proteins widely distributed among the animal kingdom that specifically recognize carbohydrates. Traditionally, they have been classified based on their ability to recognize specific carbohydrate sequences but, with the advent of new molecular biology methods, novel classes have been defined based on the presence of unique structural domains within their amino acid sequences. This novel classification stems from the presence of highly conserved carbohydrate-recognition domains (CRDs) that appear to have evolved from shared ancestral genes (1). Examples of major families of animal lectins include C-type (e.g., selectins, dectins), I-type (e.g., siglecs), P-type (mannose-6-phosphate receptors), and S-type (galectins). Among the different classes of lectins described so far, galectins have been the most extensively characterized in cornea and are the major focus of this review.

Galectins are expressed by different cell types, including epithelial, stromal, endothelial, and immune cells and typically bind β-galactose-containing glycoconjugates. They are grouped into three categories based on structure: (1) prototypical, with a single CRD that may associate to form homodimers, (2) chimeric, with a single CRD and a large amino-terminal domain that contributes to self-aggregation and, (3) tandem-repeat, with at least two CRDs occurring within a single polypeptide (2). Members of these different categories have been reported in humans and include galectins-1,−2,−7,−10,−13, and−14 (prototypical), galectin-3 (chimeric) and galectins-4,−8,−9, and−12 (tandem-repeat). Each galectin CRD recognizes distinctive carbohydrate structures in a manner that is influenced by the oligomeric state of the lectin and the multivalency of the glycan ligand (2). Galectins are exceptional in that they are synthesized on free ribosomes, exhibit no signal sequence and are secreted through a non-classical pathway that bypasses the Golgi (3). Only a few amino acids within the canonical CRD of galectins make direct contact with carbohydrate ligands, although binding sites for non-carbohydrate ligands, such as those found in the cytosol and nucleus, have also been described on the CRD. The presence of these binding domains ensures that galectins have both intracellular and extracellular activities. On the cell surface, galectins function by forming multivalent complexes with glycosylated receptors to control multiple biological events, such as receptor turnover, cell signaling, host–pathogen interactions and immune cell activation and homeostasis (4).

Other lectins that mediate biological events in cornea include selectins and dectins. Selectins are cell adhesion molecules expressed on platelets, endothelial cells, and leukocytes. They contain a single transmembrane domain and a CRD at the amino terminus with affinity toward sialylated, fucosylated structures (e.g., sialyl Lewis x) (5). Dectins are transmembrane proteins important in fungal defense expressed mainly in dendritic cells and macrophages (6). The two members of this family, dectin-1 and dectin-2, recognize β-glucans, and α-mannans, respectively.

## Structure of the cornea

The cornea is a clear, curved surface covering the anterior segment of the eye. It is responsible for refracting light onto the lens and retina in addition to resisting infection and damage. The lack of lymphatic and blood vessels is essential to maintaining the transparency of the cornea. Injury resulting from infection, transplantation, autoimmune conditions, and other pathologies can lead to the abnormal growth of vessels and loss of vision (7).

**Figure 1:**
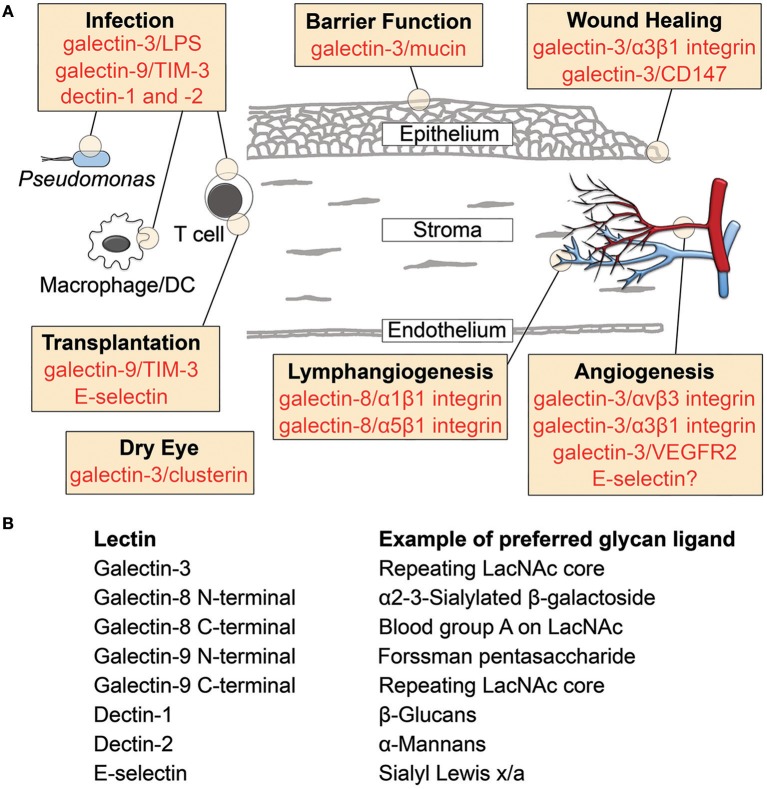
Lectin-glycan interactions reported in cornea. **(A)** Schematic diagram illustrating the involvement of lectins in cornea. Specific binding partners are indicated for galectins. **(B)** Examples of preferred glycan ligands for lectins shown in **(A)**. DC, dendritic cell; LacNAc, N-acetyllactosamine; LPS, lipopolysaccharide; TIM-3, T cell immunoglobulin- and mucin-domain-containing molecule-3.

Structurally, the cornea consists primarily of the epithelial, stromal and endothelial compartments (Figure 1). The epithelial compartment is the outermost surface and it is composed of a stratified, non-keratinized epithelium along with intraepithelial nerve terminals and dendritic cells. The stromal compartment is a dense connective tissue of significant regularity and represents the structural axis of the cornea. It is populated with keratocytes that synthesize extracellular matrix components and bone marrow derived cells that are recruited in response to injury and infection. The endothelial compartment is a simple low cuboidal epithelium that enables the exchange of ions and fluid between the stroma and the interior of the eye. The cornea is encircled by the corneoscleral limbus, which serves as a reservoir for the adult stem cell population that continuously replenishes the tissue. The use of histochemical techniques has evidenced that the cornea is rich in galectins and galectin-binding sites (8). In normal corneas, galectin-1 is present mainly in the stroma, galectin-3 localizes mainly in the epithelium, and galectins-7,−8, and−9 are present in both corneal epithelium and stroma (9).

## Lectin-glycan interactions in corneal physiology

The apical surface of the corneal epithelium constitutes an exceptional barrier against foreign particles and microorganisms that attempt to penetrate the eye. Highly glycosylated transmembrane mucins emanating from ridge-like folds of the plasma membrane are an essential component of this protective layer. They have single membrane-spanning regions with large extracellular domains that form rod-like structures, which extend over 100 nm from the cell surface, far above other glycoconjugates in the glycocalyx (10).

Research over the past decade has defined a mechanism by which transmembrane mucins contribute to the physiological protection of the corneal epithelium by interacting with galectins. Microarray analyses have revealed that the mucins MUC1 and MUC16 together with galectin-3 are among the most highly expressed glycogenes at the ocular surface (11). They localize primarily on apical membranes within the superficial stratified squamous epithelia, and the two mucins bind galectin-3 in a carbohydrate-dependent manner. Importantly, the mucin-galectin interaction is necessary to maintain galectin-3 anchored to the cell surface and to preserve transcellular barrier function in corneal epithelial cells (12). The association between transmembrane mucins and galectin-3 further functions to mask viral entry mediators on the corneal epithelial glycocalyx (13). Mechanistically, this protective function of galectin-3 is dependent on its large amino-terminal domain and the ability to form surface lattices in the epithelial glycocalyx (14).

Core 1 O-glycans are major components on transmembrane mucins at the ocular surface (15). Initial experiments targeting c1galt1, a critical galactosyltransferase required for the synthesis of core 1 O-glycans, evidenced the contribution of this modification to promoting surface retention of galectin-3 and maintaining barrier function (12). Yet, use of synthetic glycan microarrays has shown that galectin-3 displays maximum binding affinity toward N-glycans compared to O-glycans (16), implying a role for mucin N-glycans in the stabilization of the epithelial glycocalyx despite having a much lower abundance than O-glycans. Recent evidence supports this hypothesis. Structural data indicate that mucin N-glycans in cornea are rich in complex-type structures that bind galectin-3 and promote barrier integrity (17). Deciphering the relative contributions and biological significance of the different classes of mucin glycans when interacting with galectins should be an important goal of future research on mucosal surfaces.

## Lectin-glycan interactions in corneal pathology

### Ocular infection

Microbial colonization of the eye due to viral, bacterial, or fungal pathogens remains an important cause of blindness worldwide. Several findings provide strong evidence that lectin-glycan interactions play an important role in the pathogenesis and immune response to ocular infection.

Primary or recurrent episodes of herpes simplex virus (HSV) infection result in viral replication and destruction of the infected cells. This process triggers non-specific innate host defenses that contribute to infection control but also adaptive responses when dendritic cells leave the site and carry viral antigens to draining lymph nodes (18). A large number of activated T cells in ocular HSV lesions express the inhibitory molecule TIM-3 needed to control the lesion. Addition of excess galectin-9, a natural ligand of TIM-3, has been shown to diminish the severity of the lesions by inducing apoptosis of pathogenic effector Th1 cells but also increasing the representation of anti-inflammatory Tregs and decreasing neovascularization (19). Subsequent studies have shown that the interaction of galectin-9 with TIM-3 functions to constrain the response of effector and memory CD8^+^ T cells to infection (20). Other galectins, such as galectin-1, can also lessen the severity of the HSV lesion by reducing the number of IFN-γ- and IL-17-producing CD4^+^ T cells and the recruitment of neutrophils into the cornea (21).

It was the Hazlett laboratory that first reported in 1997 the presence of a member of the galectin family in cornea and its potential pathogenic contribution to bacterial infection. Using binding inhibition assays, this group found that adhesion of *Pseudomonas aeruginosa* to corneal epithelial cells could be blocked by an antibody targeting galectin-3, a binding receptor for bacterial lipopolysaccharides (22). Further work established the pattern of expression of galectins in mouse corneas under normal and infective conditions. Exposure to *P. aeruginosa* resulted in overall downregulation of galectin-3 and upregulation of galectins-8 and−9 (9). Galectin-1 within the corneal stroma appeared to limit *P. aeruginosa*-mediated inflammation by impairing the infiltration of neutrophils and CD4^+^ T cells, particularly proinflammatory Th17 cells (23).

Fungal infection is a major cause of corneal ulceration in developing countries and tropical regions commonly associated with severe inflammation. Evidence suggests that the C-type lectin receptors dectin-1 and dectin-2 play important roles in regulating disease severity and survival. Dectin-1 on corneal macrophages can be activated by β-glucans on *Aspergillus fumigatus* to promote recruitment of neutrophils into the corneal stroma and trigger fungal killing (24). Interestingly, to promote survival, *A. fumigatus* spores express RodA hydrophobin, a surface protein that confers hydrophobicity and covers cell wall components that would otherwise activate dectin-1 and dectin-2 (25). Dectin-1 also plays a critical role in cornea by controlling *Candida albicans* (26) and *Fusarium solani* (27) infections.

### Corneal transplantation

Corneas are among the most common and successful transplanted tissue worldwide. They express factors that contribute to immune privilege by inhibiting the induction and function of alloimmune T cells among others (28). Recent investigations looking at the repertoire of galectins expressed in accepted murine corneal allografts have demonstrated increased levels of galectins-1,−3,−7,−8, and−9 compared to controls (29). Interestingly, when the corneas were rejected, the levels of galectin-8 were markedly higher, whereas those corresponding to galectin-9 were substantially lower, compared to the accepted corneas. The latter complements initial observations showing that constitutive expression of galectin-9 and its ligand TIM-3 play an immunosuppressive role in corneal allografts, in particular by preventing the destruction of corneal endothelial cells by alloreactive T cells (30).

E-selectin is a carbohydrate-binding protein commonly expressed during corneal inflammatory disease (31). It localizes to vascular endothelial cells in the stroma of rejected corneal allografts, within areas with high T cell and macrophage content (32). Because of its crucial role in leukocyte extravasation and migration, E-selectin has been proposed as a therapeutic target in preventing transplant rejection. Recent data indicate that E-selectin mediates T cell recruitment in corneal transplantation and support a role for E-selectin neutralization in reducing the frequency of mature antigen-presenting cells in the draining lymphoid tissue (33). In these experiments, however, the long-term graft survival was limited, which has been attributed to the overlapping function of factors mediating leukocyte adhesion.

### Corneal injury and wound healing

Almost 40 years ago Gipson and Anderson reported the requirement of carbohydrate moieties on cell surface glycoproteins and basement membrane to promote epithelial cell migration during the healing of corneal abrasions (34). This initial work pointed to the presence of glucosamine residues on N-glycans that were upregulated as the stratified corneal epithelium became migratory (35, 36). It was not until two decades later than the Panjwani laboratory radicalized the field by implicating galectins in the re-epithelialization of corneal wounds, particularly galectins-3 and−7 (37). The molecular basis by which galectin-3 modulated epithelial migratory events included the promotion of lamellipodia formation by interacting with complex N-glycans on α3β1 integrin, and the initiation of cell-cell disassembly by inducing matrix metalloproteinase expression in a manner that was dependent on the clustering of the matrix metalloproteinase inducer CD147 (38, 39). More recently, the successful use of recombinant galectin-3 in promoting epithelial migration in non-human primate corneas has emphasized the potential of galectins as a novel therapeutic modality in wound healing (40).

It is now clear that not all kinds of injury lead to a similar expression pattern of galectins in cornea. The expression of galectin-3 is downregulated in mouse corneas following bacterial infection and chemical burn (9). Yet, galectins-7,−8, and−9 are upregulated in the epithelium following infection but not cauterization. It also appears that the changes in galectin expression during injury are species-dependent. Whereas tissue damage in mice leads to reduced galectin-3 expression, injured tissue in patients with active corneal ulceration show a greater galectin-3 immunoreactivity compared to normal subjects (41). It is possible to speculate that the inflammatory environment following injury likely influences the differential responses in galectin expression in cornea.

### Dry eye disease

Disruption of barrier function at the ocular surface is associated with a wide range of inflammatory disorders that includes dry eye, an age-related disease affecting millions of people worldwide, and whose pharmacological treatment remains unresolved. Both N- and O-glycosylation are altered in the ocular surface epithelia of dry eye patients (42), which has led to question whether there are accompanying changes in galectin expression or localization. Several studies have found that epithelial dysfunction in dry eye correlates with the release of cellular galectin-3 into tears (43, 44). This increase in extracellular galectin-3 appears to have pathological implications, since the lectin can interact with the plasma membrane of corneal epithelial cells to exacerbate the proinflammatory activities of IL-1β (45). Of particular interest are recent findings indicating galectin-3 binds to the homeostatic protein clusterin, one of the most abundant transcript in the human corneal epithelium (46). Preserving the nature of this interaction may provide therapeutic value in a variety of drying conditions at the ocular surface (47).

### Corneal angiogenesis

Corneal angiogenesis represents a major public health problem affecting 1.4 million individuals each year in the United States alone (48). The growth of new vessels occurs within the anterior corneal stroma when pro-angiogenic factors overcome anti-angiogenic stimuli. The subject of how glycosylation and galectin-3 impact vascular endothelial cells and influences corneal angiogenesis was reviewed in 2014 (49); therefore, we present a brief overview and highlight additional findings. An important breakthrough in VEGF- and bFGF-mediated angiogenesis was the discovery that galectin-3 plays a pro-angiogenic role in cornea by clustering N-glycans on αvβ3 integrin and activating focal adhesion kinase (50). This function of galectin-3 has been supported by additional data indicating that galectin-3 can activate VEGFR2 in endothelial cells (51) and form a complex with pericyte-derived NG2 proteoglycan and α3β1 integrin to promote endothelial cell motility (52). Examples of ways in which regulation of galectin-3 can have therapeutic applications have been recently described. Strategies to block galectin-3 with small-molecule inhibitors have proven efficacious in experimental models of corneal neovascularization and fibrosis (53).

In addition to galectin-3, other lectins have been implicated in corneal angiogenesis. Galectin-1 and−9 have been shown to possess anti-angiogenic activity in a mouse model of herpetic keratitis, where they decrease the production of proinflammatory cytokines and molecules involved in the formation of new vessels (19, 21). C-type lectins also appear to be critical to the process of corneal angiogenesis. Human soluble E-selectin is known to induce chemotaxis of human endothelial cells and to be angiogenic in rat cornea (54). These contributions, however, remain controversial (55). Additional experiments using corneal micropocket assays have demonstrated a role for the E-selectin cytoplasmic domain in facilitating the antiangiogenic activity of endostatin, a collagen derivative that inhibits endothelial cell migration by binding to α5β1 integrin (56). These findings evidence that formation of new vessels in cornea depends on a delicate balance of lectin-receptor interactions that can either promote or inhibit angiogenic stimuli.

### Corneal lymphangiogenesis

The lymphatic vasculature plays an important role in coordinating antigen transport and immune-cell trafficking from peripheral tissues to secondary lymphoid organs. At the ocular surface and under inflammatory conditions, lymphatics in the limbal region can give rise to new vessels that extend pathologically into the cornea (57). There is scarce information on the role of lectin-glycan interactions in corneal lymphangiogenesis, with just one report implicating galectin-8 (58). Here, the authors demonstrated that galectin-8 is markedly upregulated in inflamed corneas and can promote corneal lymphangiogenesis. Mechanistically, they found that in the absence of VEGFC or VEGFR3, the CRDs of galectin-8 crosslink integrins α1β1/α5β1 and heavily O-glycosylated podoplanin to activate lymphangiogenic signaling. These interactions can potentiate the VEGFC/VEGFR3 axis when present, and further increase the magnitude of the lymphangiogenic response.

## Concluding remarks

Progress has been made in providing mechanistic insights into the role of lectin-glycan interactions in cornea (Figure 1). Manipulating these signals represents a useful approach to control or cure ocular diseases, yet the therapeutic translation of this knowledge faces numerous challenges. For galectins, these stem from their ability to recognize a myriad of receptors on any given cell, each receptor with a unique binding affinity, in a process that is heavily influenced by the metabolic state of the cell and the cellular environment. The extent to which inhibition or activation of specific galectin signaling pathways affect others remains to be better defined, as this knowledge will be critical to produce comprehensive physiological responses. In this regard, any modulation of galectin activity will need to take into consideration the glycosylation state of the cellular receptors to achieve success. We anticipate that a better understanding of the coordinated function of lectins and glycans in cornea will unlock novel therapeutic approaches for pathological states.

## Author contributions

DA and PA reviewed the literature and wrote the article.

### Conflict of interest statement

The authors declare that the research was conducted in the absence of any commercial or financial relationships that could be construed as a potential conflict of interest.
